# Severe loss of visceral fat and skeletal muscle after chemotherapy predicts poor prognosis in metastatic gastric cancer patients without gastrectomy

**DOI:** 10.7150/jca.37270

**Published:** 2020-03-05

**Authors:** Wanjing Feng, Mingzhu Huang, Xiaoying Zhao, Siyuan Chen, Chenchen Wang, Jinjia Chang, Weijian Guo, Zhiyu Chen, Hui Zhu, Xiaodong Zhu

**Affiliations:** 1 Department of Medical Oncology, Fudan University Shanghai Cancer Center, 270 Dong' An Road, Shanghai200032, People's Republic of China; 2 Department of Oncology, Shanghai Medical College, Fudan University, 130 Dong' An Road, Shanghai 200032, People's Republic of China; 3 Department of Radiology, Fudan University Shanghai Cancer Center, 270 Dong' An Road, Shanghai200032, People's Republic of China

**Keywords:** visceral fat, skeletal muscle, metastatic gastric cancer, chemotherapy, prognosis

## Abstract

**Background**: The influence of body composition parameters in cancer prognosis attracted researchers' attention. This study investigated the role of visceral fat and skeletal muscle in the prognosis and efficacy of chemotherapy in metastatic gastric cancer (MGC).

**Methods**: This study included MGC patients without gastrectomy treated with EOF regimen (epirubicin, oxaliplatin and fluorouracil), who participated in a Phase II clinical trial (NCT00767377) with available PACS image data. The visceral fat area (VFA) and skeletal muscle area (SMA) were measured using standard computed tomography (CT).

**Results**: A total of 46 patients were enrolled in the study. Patients with low baseline VFA and SMA had significantly shorter PFS and OS. In addition, the loss of VFA and SMA also predicts significantly shorter PFS and OS. A prognostic index that included two risk factors, severe loss of VFA and SMA, was used to categorize the patients into two groups: good-risk group (0 risk factors), poor-risk group (1 or 2 risk factors). Compared with the good-risk group, the poor-risk group displayed a 3.562-fold-increased risk of progression [hazard ratio (HR) 3.652, 95 % CI 1.653-7.678; P =0.001] and 2.859-fold-increased risk of death [hazard ratio (HR) 2.859, 95 % CI 1.271-6.434; P =0.011].

**Conclusion**: Low baseline VFA and SMA, as well as the severe loss of VFA and SMA predict poor prognosis for MGC patients treated by EOF regimen. In patients with severe loss of VFA and/or SMA after 2-cycle chemotherapy, the decision of subsequent chemotherapy should be made after deliberate consideration.

## Introduction

Gastric cancer (GC) is the fourth most common malignant neoplasm and the second leading cause of cancer-related death worldwide 1. Unfortunately, the response rate of GC to chemotherapy is low. Even with intensive first-line treatment regimens, such as ECF (epirubicin, cisplatin and fluorouracil), EOF (epirubicin, oxaliplatin and fluorouracil), and EOX (epirubicin, oxaliplatin and capecitabine), the response rate is still <50% in patients with metastatic GC (MGC). Although some patients benefit from chemotherapy, the side effects are unavoidable. Therefore, biomarkers that can predict chemotherapeutic efficacy are urgently needed.

Recently, obesity and sarcopenia are identified as prognostic factor in colorectal cancer 2, 3 and hepatocellular carcinoma^4^, ^5^. Recent evidence suggests that visceral fat plays a key role in carcinogenesis rather than general body fat 6, 7, 8, 9, 10. Thus, visceral fat might more accurately measure carcinogenesis caused by adipose tissue than body mass index (BMI). Sarcopenia is the progressive and systematic loss of skeletal muscle with atrophy 11 and recognized as a risk factor for physical disability, decreased quality of life and ultimate death.

Therefore, visceral fat area and skeletal muscle area measured by computed tomography (CT) are proposed as body composition factors. CT is a “golden standard” to measure fat 12 and muscle area 13. These body composition factors have been evaluated in different kinds of tumors 14-17. In terms of gastric cancer, low visceral fat has been reported as a poor prognostic factor in GC patients with surgery resection 18. Besides, the loss of visceral fat and skeletal muscle are also reported to predict poor prognostic factor in patients with who underwent gastrectomy 19.

However, no research has investigated whether visceral fat and skeletal muscle can predict prognosis and chemotherapy response in patients with MGC who lose the chance of surgery. The aim of this retrospective study was to reveal the association between body composition parameters measured by computed tomography (CT) and chemotherapy response in patients with MGC.

## Materials and Methods

The current study was a retrospective analysis based on a Phase II clinical trial (NCT00767377) that was conducted at Fudan University Shanghai Cancer Center during June 2007 to July 2012. Due to equipment updates, the new PACS CT system only contains image data after January 2010. Patients with resection of primary lesion and incomplete image data were excluded. All the enrolled patients were confirmed with unresectable MGC and treated with EOF regimen as first-line chemotherapy. Their diagnosis was certified by pathological examination as gastric adenocarcinoma. The specific therapeutic regimen was as described previously 20. Tumor responses were evaluated in accordance with the Response Evaluation Criteria in Solid Tumors 1.0 (RECIST 1.0) by contrast-enhanced CT scans every two cycles 20.

### Measurement of VFA and SMA by CT

CT images were obtained with multidetector row CT scanners (Somatom Sensation 40 or Somatom Sensation 64; Siemens AG, Medical Solutions, Business Unit CT, Forchheim, Germany), with a slice thickness of 5 mm. Images were uploaded to a picture archiving and communication system (PACS, GE Healthcare-Centricity RIS CE V2.0, GE Medical Systems, US). CT image analysis was performed with the image post-processing software module (AW module) embedded in this PACS. We selected the cross-sectional CT images at the third lumbar vertebra (L3) as a standard landmark to quantify visceral fat area (VFA) and skeletal muscle area(SMA) according to previous literatures 17, 21. Two consecutive cross-sectional CT images at L3 level were reformatted by AW module. Preset thresholds of Hounsfield units (HU) were as follows: -190HU to -30HU for fat tissue, and -30HU to 90HU for skeletal muscle tissue. Total abdominal fat volume (including visceral fat and subcutaneous fat) and skeletal muscle volume of selected two consecutive slices were quantified automatically by AW module. Then, we obtained visceral fat volume by removing subcutaneous fat from total abdominal fat manually. Finally, we could calculate VFA and SMA by dividing these measurements by thickness (two consecutive images, 5mm×2 =1cm).

### Statistical analysis

Kaplan-Meier survival was used to estimate progression-free survival (PFS) and overall survival (OS) of the four quartiles. Receiver operating characteristic (ROC) curve was used to establish a cut-off value of visceral fat area (VFA) and skeletal muscle area (SMA) to distinguish patients who were expected to have longer PFS and OS from those who were not. Survival curves were estimated by Kaplan-Meier method and analyzed by log-rank test. A multivariate Cox proportional hazards model was used to evaluate the role of VFA and SMA in predicting PFS and OS, after adjusting for clinical characteristics, including age, gender, liver metastasis, lung metastasis, ascites and/or pleural effusion. We use VRV to indicate variation rate of visceral fat area at baseline and after 2-cycle chemotherapy. VRS was used to stand for variation rate of skeletal muscle area at baseline and after two cycles' chemotherapy. Statistical analysis was conducted using Stata version 12.0 software (StataCorp LP, College Station, TX, USA). P<0.05 (two-sided) was considered significant.

## Results

### Patient characteristics

In the new PACS system, image data of baseline and after two-cycle treatment were available only in 53 patients. Among these patients, a total of 46 patients were enrolled in the study after exclusion of patients with gastrectomy. The characteristics of the 46 enrolled patients are shown in Table 1. The median PFS and OS of the 46 patients were 6.0 months [95% confidence interval (CI) 4.9-7.1] and 19.0 months (95% CI 10.7-27.3), respectively, which were slightly different form the PFS and OS results of the total 150 patients reported previously (6.0 months (95% CI 5.4-6.6) and 12.6 months (95% CI 8.2-16.9), respectively) 20.

### Baseline VFA/SMA and clinical outcome

**Figure 1:**
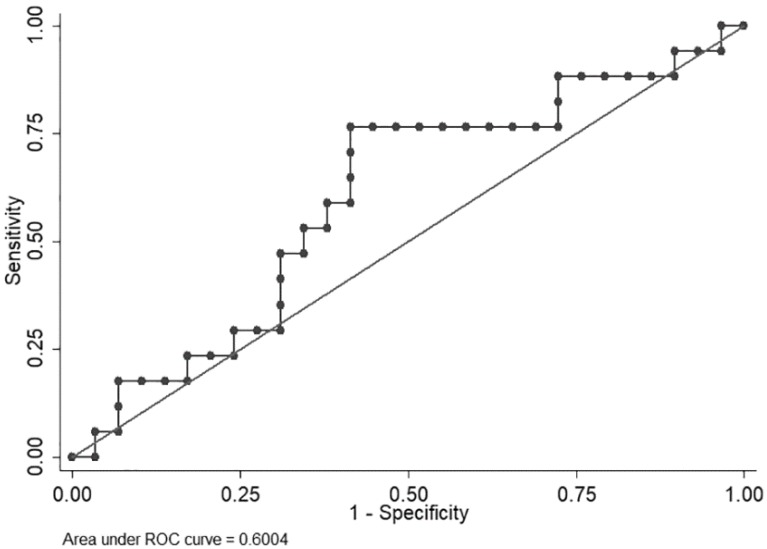
ROC curve of visceral fat area to identify patients with longer progression-free survival time.

VFA exhibited large variations; therefore, an ROC curve was used to identify a cut-off level to discriminate patients who were expected to have disease progression within a short period (<6.0 months) from those who were not. Median PFS in the clinical trial (NCT00767377) was 6.0 months. The area under the curve (AUC) was 0.6004. A VFA value of 62.656 cm^2^ offered the best overall sensitivity (76.47%) and specificity (58.62%) (Figure 1).

**Figure 2:**
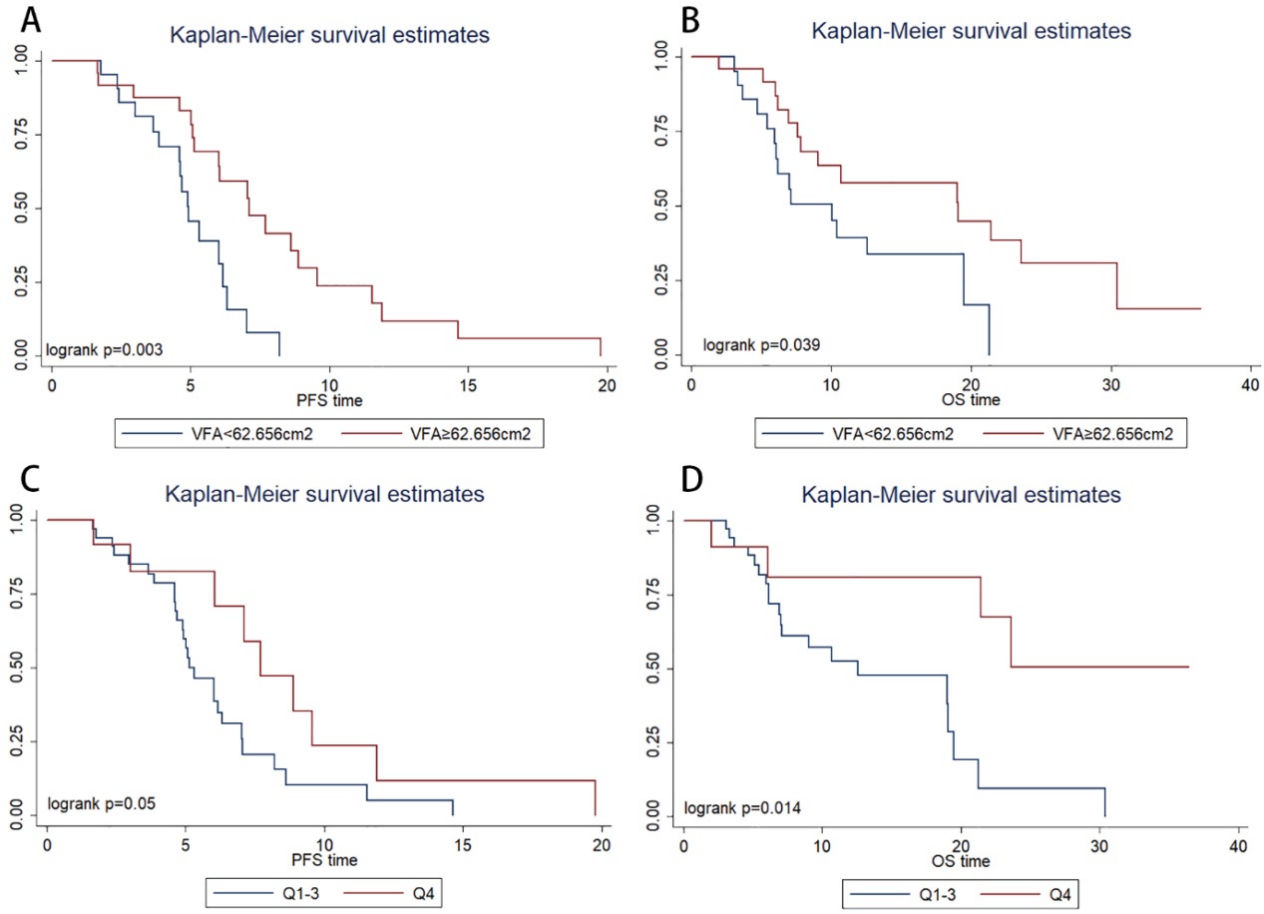
Kaplan-Meier progression-free survival(A) and overall survival(B) curve in patients with different value of baseline visceral fat area (VFA). Kaplan-Meier progression-free survival(C) and overall survival(D) curve in patients with different baseline skeletal muscle area.

Kaplan-Meier survival estimates showed that patients with VFA>62.656 cm^2^ had significantly longer PFS (*P*=0.003, 4.9 vs. 7.1 months) and OS (*P*=0.039, 12.5 vs. 21.4 months) than those with VFA≤62.656 cm^2^ (Figure 2A, Figure 2B, respectively). In multivariate Cox regression analysis, VFA>62.656 cm^2^ was significantly associated with longer PFS (hazard ratio, 0.160; 95% CI, 0.048-0.532; *P*=0.003) and OS (hazard ratio, 0.294; 95% CI, 0.091-0.945; *P*=0.040) (Table 2). The objective response rate (ORR) were 60% in VFA>62.656 cm^2^ group and 38% in VFA≤62.656 cm^2^. ORR was not significantly associated with VFA (*p*=0.236).

However, an ROC curve failed to identify a cut-off level of SMA to discriminate patients who were expected to have disease progression within a short period (<6.0 months) from those who were not. The patients were categorized into four quartiles (Qs) based on baseline SMA (Q1, ≤ 99.1 cm^2^; Q2, 99.1 to 118.3 cm^2^; Q3, 118.3 to 133.9 cm^2^; and Q4, ≥133.9 cm^2^ ). In the Kaplan-Meier analysis, the high SMA group (Q4) had a significantly longer PFS (*P*=0.050, Figure 2C) and OS (*P*=0.014, Figure 2D) than the low SMA group (Q1-3). ORR was not significantly associated with SMA (*P*=0.738).

### Severe loss of VFA/SMA and clinical outcome

**Figure 3:**
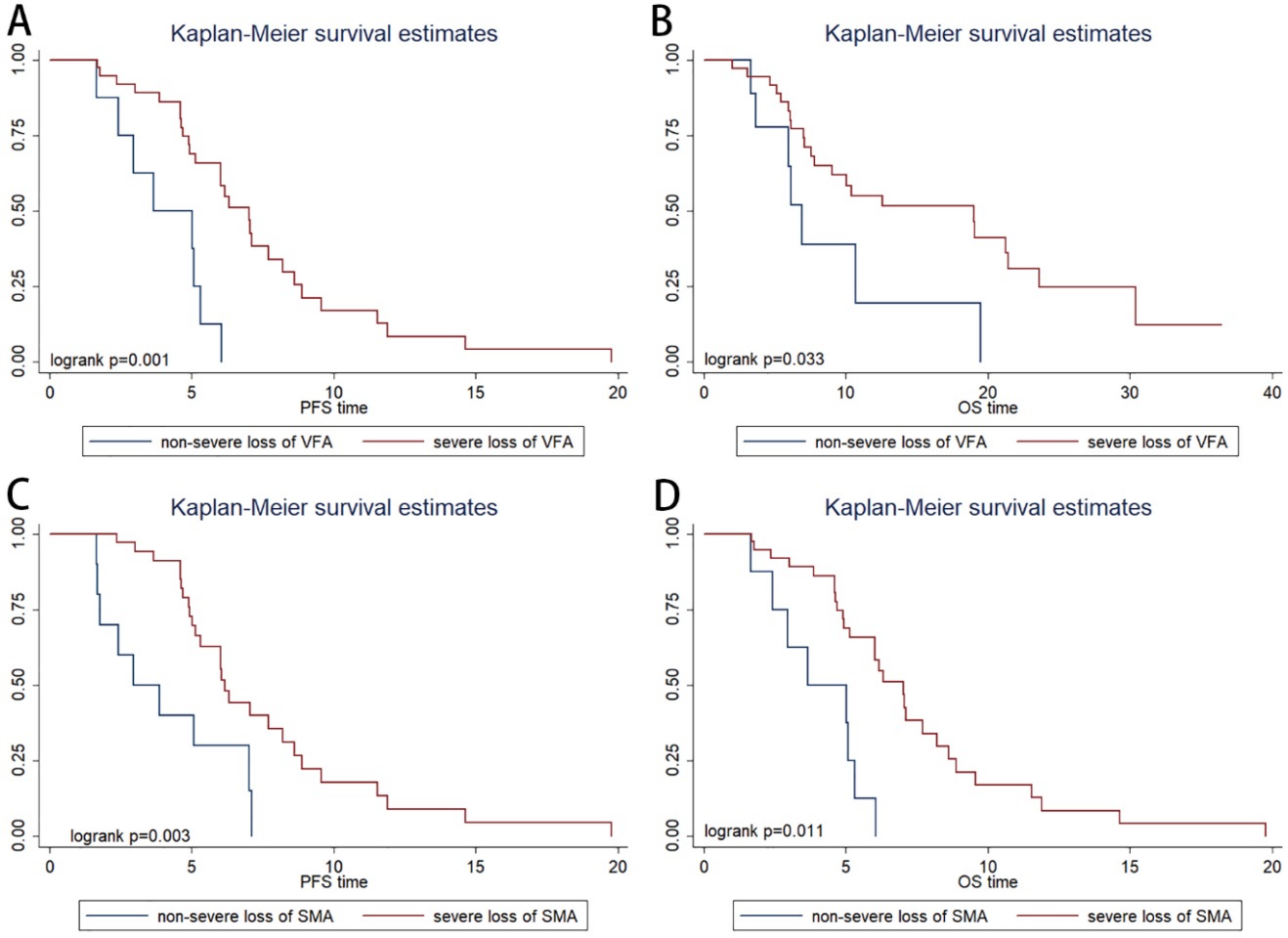
Kaplan-Meier progression-free survival (A) and overall survival (B) curve in patients with different variation rate of visceral fat area. Kaplan-Meier progression-free survival (C) and overall survival (D) curve in patients with different variation rate of skeletal muscle area.

The patients were categorized into four quartiles (Qs) based on variation rate of VFA (VRV) (Q1, ≤ -20%; Q2, -20% to 0%; Q3; 0% to 29%; and Q4; >29%). In the Kaplan-Meier analysis, severe VFA loss group (Q1; patients with visceral fat loss ≥ 20%) had a significantly shorter PFS than non-severe VFA loss group (Q2-4; patients with visceral fat loss<20% or with visceral fat increase) (*P*=0.001, Figure 3A) and OS (*P*=0.033, Figure 3B). ORR was not significantly associated with VRV (*P*=0.491).

The patients were categorized into four quartiles (Qs) based on variation rate of SMA (VRS) (Q1, ≤ -8%; Q2, -8% to -3%; Q3; -3% to 2%; and Q4; >2%). In the Kaplan-Meier analysis, severe SMA loss group (Q1; patients with muscle loss ≥ 8%) had a significantly shorter PFS (*P*=0.003, Figure 3C) and OS (*P*=0.011, Figure 3D) than non- severe SMA loss group (patients with muscle loss<8% or with muscle increase). ORR was not significantly associated with VRS (*P*=0.738).

### Combination analysis of Severe loss of VFA and SMA

**Figure 4:**
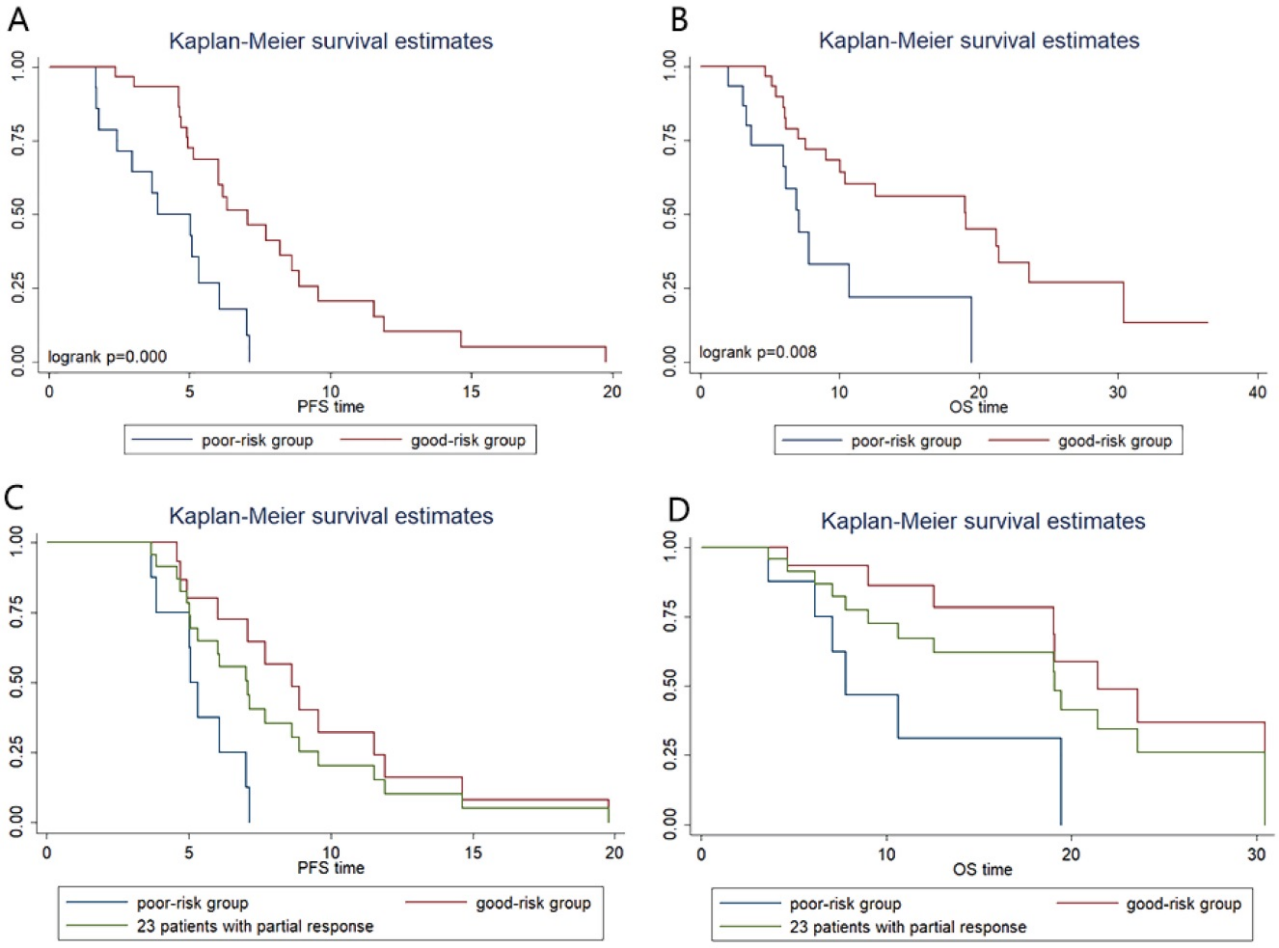
Kaplan-Meier progression-free survival and overall survival curve according to prognostic index. (A) and (B) show differences in PFS and OS, respectively. In patients with partial response, Kaplan-Meier progression-free survival (C) and overall survival (D) curve according to prognostic index.

The severe loss of VFA or SMA after chemotherapy was considered as two risk factors and we did a combination analysis of the two factors. Patients were categorized into two groups: the good-risk group (n=31), patients with no risk factors; and the poor-risk group (n=15), patients with one or two risk factors. We found that poor-risk group had significantly shorter PFS (3.8m vs 7.0m;* P*=0.000, Figure 4A) and OS (7.1m vs 19.0m;* P*=0.008, Figure 4B) than patients in good-risk group. In multivariate Cox regression analysis, severe loss of VFA and/or SMA is an independent predictive factor for shorter PFS (hazard ratio, 0.325; 95% CI, 0.134-0.788; *P*=0.013) and OS (hazard ratio, 0.352; 95% CI, 0.137-0.903; *P*=0.030) (Table 3). Compared with the good-risk group, the poor-risk group displayed a 3.562-fold-increased risk of progression [hazard ratio (HR) 3.652, 95 % CI 1.653- 7.678; *P*=0.001] and 2.859-fold-increased risk of death [hazard ratio (HR) 2.859, 95% CI 1.271-6.434; *P*=0.011].

In addition, we performed further analysis in patients with partial response (PR) to chemotherapy (Figure 4C, Figure 4D). In PR patients, whose tumor retraction were favorable to chemotherapy, the median OS of patients with poor risk was only 7.8 m, which was significantly shorter than that of patients with good-risk (21.4m, *P*=0.01) and shorter than that of the 46 patients (19.1m).

## Discussion

We found that low baseline visceral fat area and skeletal muscle area were both significantly associated with shorter PFS and OS in MGC patients treated with EOF regimen. Moreover, the multivariate risk factor model confirmed that VFA was an independent predictive factor for PFS and OS. Baseline VFA and SMA, like Karnofsky score, reflects patients' nutrition status and performance status. Good visceral fat and skeletal muscle reserves indicate that the patients are in good physical condition to receive chemotherapy.

We found severe loss of VFA or SMA is associated with shorter PFS and OS. Then we combined the two risk-factors of severe loss of VFA and SMA to create a risk model, and our data confirmed that severe loss of VFA or SMA was an independent predictive factor for PFS and OS. For severe loss of VFA and/or SMA are the individualized responses of each patient to specific chemotherapy regimen, our risk model is helpful in judging patients' tolerance to chemotherapy, trends in disease development, prognosis, and clinical benefit.

One more interesting point of our findings is that we found our risk model is more precise in evaluating further clinical benefit than chemotherapeutic response. It is proven that patients with partial response (PR) to chemotherapy have better PFS and OS than patients with stable disease (SD) or with progression disease (PD) 22. PR is considered as a good prognostic factor, and patients with PR is always believed to get benefit from chemotherapy. However, our result indicated that even in patients with partial response, the median OS of those with severe loss of VFA or SMA was significantly shorter than that of those without (7.8 months versus 21.4 months). The huge difference suggested that for those patients the survival benefit of further chemotherapy was rather limited. The median number of the courses of chemotherapy in patients with severe loss of VFA/SMA was 6(5-8), and the number in patients without severe loss of VFA/SMA was 6(5-8). It means that there is no significant difference in the chemotherapy intensity between the two groups, which exclude the possibility that the difference in survival between the two groups was due to the reduction of subsequent chemotherapy intensity in patients with severe loss of VFA/SMA. Therefore, for those patients, improvement of the poor nutritional status may be more important than chemotherapy. In this situation, the decision of whether patients still need subsequent chemotherapy should be made after deliberate consideration.

These composition parameters, including VFA, SMA, VRV and VRS, can be used together with performance status and tumor biomarkers, and help us to make clinical decisions. In patients with difficulties in evaluating efficacy after two-cycle chemotherapy, these parameters are especially useful for oncologists to evaluate whether patients will benefit from subsequent chemotherapy.

The composition parameters are closely related to malnutrition. Malnutrition may affect the tolerance of chemotherapy, quality of life and survival 23, 24. Cachexia, which is a life-threatening condition and observed in 85% of gastric cancer patients 25, accounts for more than 20% of cancer death 26, 27. Cachexia is composed of the loss of skeletal muscle and adipose tissue. Sarcopenia is observed in different kinds of cancer, which is associated with prognosis. Cancer cells produce inflammatory cytokines, resulting in systematic inflammation. These inflammatory cytokines may trigger muscle wasting and skeletal muscle atrophy, resulting in sarcopenia 26, 28. The loss of skeletal muscle is also considered to predict poor prognosis in colorectal cancer 14 and gastric cancer 19.

Visceral fat is considered as a poor prognostic factor for survival in colorectal cancer 29-33 and pancreatic cancer 34. However, in our study, low visceral fat and the loss of visceral fat predicts significant poor prognosis, which is supported by previous studies. Hyung S.P. et al conducted a single-center retrospective study from the CLASSIC Trial and revealed that the marked loss of visceral fat predicts shorter DFS and OS in GC patients who underwent gastrectomy 19. Another retrospective study conducted by Kazuto H. showed that patients with upper gastrointestinal cancer with low visceral fat content had shorter OS than those with high visceral fat content 18. We suggest three reasons for the opposite results in gastric cancer that low visceral fat predicts poor prognosis. First, all the enrolled patients are in stage IV, the oral ingestion disorder of which is poorer than patients in early stage. Nutritional deficiency can be caused by decreased intake, resulting in cachexia, which is associated with poor cancer prognosis. Second, fat is an energy reserve and loss of fat is a part of nutritional deficiency. Patients with better nutritional status and energy reserves are expected to live longer. Third, the loss of visceral fat can be considered as the process of energy consumption. The more energy is consumed, the shorter lifetime is expected.

In our study, patients who underwent the resection of primary lesion were excluded and there are two reasons. First, gastrectomy might remove the visceral adipose tissue, which affects the value of baseline visceral fat area before chemotherapy. Second, patients with gastrectomy lost the majority or the total of their stomach, which has a strong impact on nutrition absorption. All the researches that we have mentioned above focused on the GC patients in early stage who have underwent surgery with/without adjuvant chemotherapy. Therefore, the results may reflect the influence of both surgery and chemotherapy. Our study excluded all the patients with gastrectomy and focus on the influence of chemotherapy in the change of visceral fat and skeletal muscle. To the best of our knowledge, this is the first time that the composition parameters, visceral fat and skeletal muscle, are analyzed in MGC patients who lost the chance of the surgical resection at the initial diagnosis to predict prognosis and chemotherapy response.

Actually, compared with previous studies 15, 19, 35, 36, our study has several novelties. First, our study merely included metastatic gastric cancer who did not undergo gastrectomy, which exclude the influence of surgery in the content of visceral fat/skeletal muscle, which is different from previous studies. Second, our study established a new prognostic index, in which patients who had severe loss of visceral fat and skeletal muscle after chemotherapy had significantly shorter overall survival. Thus, we believe in patients who had severe loss of visceral fat and skeletal muscle after chemotherapy, the subsequent chemotherapy might not be suggested.

Our study also had some limitations. First, the sample size was small. Because of equipment replacement in our center, only one third of patients in the Phase II trial were enrolled in this study. Second, the CT scans were evaluated by only one experienced radiologist, which might have caused deviation. Third, we do not have a validation population to confirm our results. Actually, we have tried our best to search for an independent cohort to verify our results. However, the public database we have searched, including TCGA, SEER, ICGC, and GEO, have no information about abdominal fat content or skeletal muscle content. We have conducted a similar research in an ongoing phase III clinical trial in our center and try to verify our results.

In conclusion, VFA measured by CT scans can be used as a predictive factor for PFS and OS in MGC patients treated with EOF regimen. The severe loss of visceral fat and skeletal muscle can be used to predict shorter PFS and OS. In patients with severe loss of VFA and SMA after 2-cycle chemotherapy, the decision of subsequent chemotherapy should be made after deliberate consideration.

## Figures and Tables

**Table 1:** Clinicopathologic characteristics of 46 patients.

Characteristics	Number of Patients
**Sex**	
Male	29(63.0%)
Female	17(37.0%)
**Age (year)**	
≥60	30(65.2%)
<60	16(34.8%)
**ECOG**	
0	3(6.5%)
1	42(91.3%)
2	1(2.2%)
**Liver Metastasis**	
Yes	20(43.5%)
No	26(56.5%)
**Lung Metastasis**	
Yes	4(8.7%)
No	42(91.3%)
**retroperitoneal lymph nodes**	
Yes	20(43.5%)
No	26(56.5%)
**ascites**	
Yes	12(26.1%)
No	34(73.9%)
**hydrothorax**	
Yes	5(10.8%)
No	41(89.2%)

**Table 2:** Multivariate Cox regression analysis of prognostic factors for progression-free survival and overall survival (visceral fat area as categorized variate).

Clinical characteristics	Progression-free survival		Overall survival	
	Hazard ratio(95% CI)	p	Hazard ratio(95% CI)	p
Gender	4.010(0.940-17.117)	0.061	0.881(0.184-4.218)	0.874
Age	1.060(1.001-1.123)	0.046	1.027(0.970-1.088)	0.360
Liver metastasis	0.336(0.140-0.806)	0.015	0.318(0.121-0.837)	0.020
Lung metastasis	0.408(0.066-2.509)	0.333	0.822(0.126-5.373)	0.838
Ascites and/or pleural effusion	0.261(0.087-0.784)	0.017	0.274(0.074-1.015)	0.053
Visceral fat area	0.160(0.048-0.532)	0.003	0.294(0.091-0.945)	0.040
Skeletal muscle area	1.017(0.991-2.509)	0.199	0.999(0.970-1.029)	0.938

**Table 3:** Multivariate Cox regression analysis of prognostic factors for progression-free survival and overall survival.

Clinical characteristics	Progression-free survival		Overall survival	
	Hazard ratio(95% CI)	p	Hazard ratio(95% CI)	p
Gender	2.581(0.998-6.678)	0.051	1.119(0.426-2.941)	0.820
Age	0.549(0.249-1.209)	0.137	0.777(0.353-1.709)	0.530
Liver metastasis	0.398(0.157-1.009)	0.052	0.372(0.138-1.003)	0.051
Lung metastasis	0.666(0.141-3.148)	0.608	0.471(0.097-2.296)	0.352
Ascites and/or pleural effusion	0.416(0.161-1.076)	0.070	0.400(0.133-1.207)	0.104
Severe loss of VFA and/or SMA	0.325(0.134-0.788)	0.013	0.352(0.137-0.903)	0.030
